# Does pelvic asymmetry in children is related to pelvic asymmetry of their parents?

**DOI:** 10.1186/1748-7161-10-S1-P14

**Published:** 2015-01-19

**Authors:** Maciej J Dluski

**Affiliations:** 1 ART- Centre of Natural Therapies, Rzeszow, Poland

## Objectives

Asymmetry within the pelvic structure can lead to a cascade of postural compensations throughout the spine, predisposing people to recurrent somatic dysfunction and decreased functionality. As a basic structural element, the pelvis should be fully symmetric with respect to the sagittal plane. However, many studies conducted by different researchers showed, that the asymmetry of the pelvis is a very common case. This asymmetry was recorded as a result of various types of measurements made on the basis of X-rays, TK as well as sectional preparations. The reason for this was seen in the shortened one of the lower limbs or the lumbar spine pathology.

The aim of this paper is to answer the question: If there is any relationship between pelvic asymmetry in children and pelvic asymmetry of their parents?

## Material and methods

**Figure 1:**
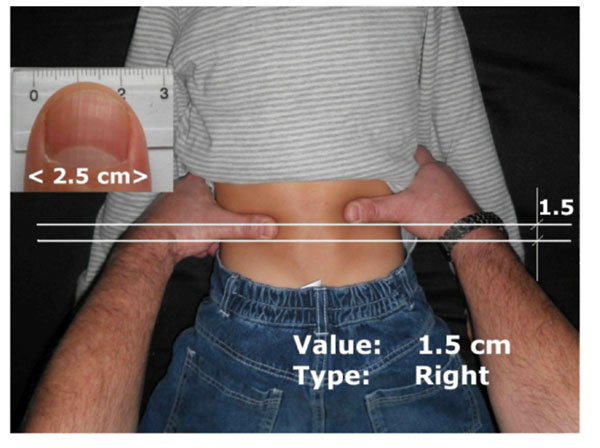
The method of measurements (child's pelvic)

A total of 180 volunteer subjects were included in the study and classified into 60 groups. Each group included one child (aged 3-17 years, 30 males and 30 females) and its biological parents; mothers aged 29 to 50 and fathers aged 30 to 52. To determine the asymmetry of the pelvis, each person was tested in the same manner. The palpation examination of anatomical landmarks (the highest points of iliac crest) has been carried out in a symmetrical prone position of the tested one. Approved measurement accuracy was 0.5 centimeters. Type of asymmetry marked as the term, which iliac crest was more distant from the investigator. Method of the study is shown in a Figure 1.

## Results

The results of measurements of pelvic symmetry are presented in Tables 1 and 2.

**Table 1:** Measurement of the pelvis (type). Number and percentage of cases

Type	Child		Mother		Father	
Neutral	7	11%	8	13%	28	46%

Left *	2	3%	0	0%	1	2%

Right *	51	86%	52	87%	31	52%

* iliac crest more distant from the investigator

**Table 2:** Correlation between pelvic asymmetry/symmetry in children and their parents (value and type)

Child	Mother	Father
Value	0.65	-0.24

Type	0.93	-0.34

Marked correlations are significant at p < 0,05

## Conclusions

The carried out research shows that there is a relationship between the laying of the pelvic bones of the child and mother. Correlation of the measured displacement is significant and amounts to 0.65. In terms of type of asymmetry is a very strong correlation and its value is equal to 0.93. The compound of symmetry of the pelvis of the child and father is negligible. Although the correlation is negative (respectively - 0.24 and - 0.34, inverse correlation), the strength of this correlation is small and irrelevant. The results encourage further exploration. The problem is still open.

## Consent

Written informed consent was obtained from the patient for the image(s) used in this study. A copy of the written consent is available for review by the Editor of this journal.

## Competing interests

The author declare that they have no competing interests.

